# Assessing Zoonotic Disease Exposure and Occupational Health and Safety Practices Among Veterinary Services Fieldworkers in North West Province, South Africa

**DOI:** 10.3390/ijerph22101577

**Published:** 2025-10-16

**Authors:** Sboniso Mhlongo, Nisha Naicker, Tanusha Singh

**Affiliations:** 1 North West Department of Agriculture and Rural Development, Veterinary Services, Potchefstroom 2520, South Africa; smhlongo@nwpg.gov.za; 2 Department of Environmental Health, Faculty of Health Sciences, University of Johannesburg, Doornfontein 2028, South Africa; nishan@nioh.ac.za; 3 A Division of the National Health Laboratory Services, National Institute for Occupational Health, Braamfontein 2001, South Africa

**Keywords:** zoonotic diseases, occupational exposure, veterinary fieldworkers, personal protective equipment, South Africa, occupational health and safety

## Abstract

Background: Veterinary fieldworkers play an important role in managing animal and public health risks, yet they face significant occupational hazards, particularly from zoonotic diseases. In South Africa’s North West Province, the occupational health and safety (OHS) of this workforce remains understudied. This study aimed to describe and characterize the OHS practices, zoonotic disease risk exposures, and contributing factors affecting veterinary services fieldworkers in the North West Province. Methods: A cross-sectional descriptive study was conducted among 137 veterinary fieldworkers, including animal health technicians, state veterinarians, and veterinary public health officers. Data were collected using a structured, self-administered questionnaire focusing on sociodemographics, knowledge of zoonoses, exposure history, and OHS practices. Descriptive statistics were applied using SPSS version 27. Results: Fieldworkers frequently reported contact with animals, animal waste, and body fluids without consistent access to adequate personal protective equipment (PPE) or comprehensive training. While most were aware of common zoonoses such as rabies and brucellosis, less than half received regular OHS training or vaccinations. Significant associations were found between occupational category and reported PPE use, as well as between knowledge levels and years of experience. Conclusions: This study highlights critical gaps in knowledge of zoonotic disease, inconsistent implementation of safety protocols, and inadequate PPE provision and/or use among veterinary fieldworkers. These findings highlight the urgent need to strengthen occupational health frameworks, standardize training, and improve access to protective resources to safeguard both workers and broader public health.

## 1. Introduction

Zoonoses are diseases or infections that are naturally transmitted from animals to humans [1]. Their incidence has risen alarmingly, with over 30 new human pathogens emerging between 1980 and 2022 [2], and more than 75% of these pathogens originating from animals [3]. According to the Centers for Disease Control and Prevention [4], six out of every ten infectious diseases in humans are zoonotic, emphasizing the urgent need for responsive national health systems. The emergence and re-emergence of zoonotic diseases are driven by several factors, including environmental changes, microbial adaptation, and human activities that disrupt the human–animal interface [4]. As a result, close human contact with animals, whether in agricultural, domestic, or natural settings, poses significant public health challenges globally [5].

In occupational environments where routine human–animal interactions occur, the risk of exposure to zoonotic hazards is significantly elevated [6]. This highlights the importance of occupational health, a core branch of public health that aims to promote and protect workers’ physical, mental, and social well-being [7]. Globally, around 49% of the population is engaged in agricultural activities, often involving animal husbandry and exposure to zoonotic risks [6]. Among these, veterinary service workers, such as state veterinarians, animal health technicians (AHT), and support staff, face particularly high risks due to their frequent contact with infected animals and contaminated biological materials [8]. Consequently, it is critical that occupational health protocols in veterinary settings incorporate both biosafety and biosecurity practices to ensure the safe handling, storage, and disposal of infectious materials and to prevent both unintentional and intentional release of pathogens [9].

Biosecurity in animal-handling facilities refers to measures aimed at preventing the entry and exit of pathogens, thereby protecting animal health, human health, and food safety [10,11]. For veterinary fieldworkers, knowledge and consistent implementation of biosafety and biosecurity principles, such as correct use of personal protective equipment (PPE), routine disinfection, changing gloves, hand hygiene, and minimizing personnel movement in areas of infection, are essential to reduce the spread of disease between animals and to ensure both worker and animal safety [10,11].

Several studies have demonstrated elevated risk among veterinary personnel, especially those working in farms and abattoirs, who are up to three times more likely to contract zoonotic diseases compared to other occupational groups [8]. In South Africa, a study in Mpumalanga Province revealed that 95% of workers in animal-related jobs tested positive for at least one zoonotic disease [12], while 63.3% of veterinarians at the University of Pretoria, Gauteng Province, reported having previously contracted a zoonotic infection [8]. These patterns are particularly concerning, as increased infection rates can enable animal pathogens to adapt for human-to-human transmission, resulting in disease outbreaks.

This situation is further aggravated by gaps in occupational health knowledge, awareness, and preventive practices. For instance, a study from the United States of America (USA) found that only 37% of veterinarians used PPE when handling sick animals, and 43% were unaware of disease reporting protocols [13]. This was associated with fewer years of professional experience, which influenced knowledge, attitudes, and occupational practices [13]. Similarly, in the United Kingdom (UK), only 31% of veterinary practices had staff trained in health and safety procedures, contributing to low awareness of occupational health protocols [14]. In Durban, South Africa, just 9.3% of veterinary professionals surveyed were aware of biological waste disposal regulations, resulting in unsafe practices and elevated exposure to zoonotic agents [15].

Based on these risks, the World Health Organization (WHO) has recommended preventive measures such as vaccination, for example, pre-exposure prophylaxis for rabies, which has been adopted in South Africa [16,17]. However, research suggests that no additional vaccines for occupationally significant zoonoses have yet been formally adopted in the country.

The selection of diseases and pathogens was informed by the province’s surveillance programmes and their relevance to occupational exposures [18]. The findings are intended to guide future interventions to reduce occupational zoonotic disease risk and enhance the safety and resilience of veterinary fieldworkers.

## 2. Materials and Methods

### 2.1. Study Design and Setting

A cross-sectional quantitative study was conducted to assess the OHS knowledge, awareness, and practices of veterinary services fieldworkers in the North West Province of South Africa. The study was carried out within the Directorate of Veterinary Services under the Department of Agriculture. The North West Province was selected for its large animal health workforce compared to other provinces and high zoonotic risk, with over 60% of controlled animal disease outbreaks reported in 2021 attributed to zoonotic origins [18].

### 2.2. Study Population

Fieldworkers included in this study were those who, as part of their routine duties, were regularly (i.e., at least monthly) exposed to animals and/or their biological products. The target population comprised all 137 field-based employees of the Directorate of Veterinary Services, including three primary occupational categories (Table 1): state veterinarians (Vets), animal health technicians (AHTs), general/farm aid workers, and veterinary public health officers (VPHOs). These roles were included due to their direct involvement in animal and public health functions that entail potential zoonotic exposure.

### 2.3. Sampling and Recruitment

A census approach was employed to include all 137 eligible veterinary services fieldworkers. Invitations to participate were sent via email to the Director and Managers of the different districts, who in turn distributed them to the officials. The invitation included a detailed information sheet outlining the study’s purpose, procedures, and ethical considerations, along with a consent form. Participation was voluntary, and no incentives were provided. Respondents were given the option to complete either an electronic or a printed version of the questionnaire, depending on their preference and access.

### 2.4. Data Collection

Data were collected using a self-administered structured questionnaire developed using Microsoft Forms (Microsoft Corporation, Redmond, WA, USA). The questionnaire was piloted on ten individuals, who were later excluded from the main study, as they were no longer employed under the Directorate, which was a criterion for inclusion. The pilot study produced a Cronbach’s alpha (α) value exceeding 0.7, indicating that the instrument was both reliable and consistent. Each respondent took approximately 15–20 min to complete the survey. The questionnaire was administered in English, as it is the language spoken among the fieldworkers. The questionnaire consisted of four sections: (1) sociodemographic characteristics, (2) OHS knowledge and practices, (3) workplace risk factors and zoonotic exposure history, and (4) perceptions and recommendations for OHS improvements to inform future interventions and policy reforms. The questionnaire was underpinned by a conceptual framework positing that OHS knowledge and awareness influence individual workplace practices and attitudes toward risk mitigation [19]. Key components assessed included the following:

- Internal training (e.g., departmental induction, in-house OHS training, and specific biosecurity or biosafety modules);
- External training (e.g., academic coursework, workshops from previous employers);
- Ability to distinguish between zoonotic and non-zoonotic diseases;
- Frequency and adequacy of PPE usage;
- Perceived gaps in departmental OHS systems and processes.

### 2.5. Data Management

Survey data from both electronic and hardcopy submissions were collated, cleaned, and coded using Microsoft Excel (Microsoft Corporation, Redmond, WA, USA). The dataset was subsequently exported into IBM SPSS version 27 (IBM Corporation, Chicago, IL, USA) for statistical analysis. The majority of the data were automatically exported into an Excel spreadsheet from Microsoft Forms, while manually captured data were cross-verified against the number of hard-copy responses to prevent duplicate entries.

### 2.6. Statistical Analysis

Descriptive statistics, frequencies, and percentages were used to summarize categorical variables. Responses to knowledge questions were scored as “yes” or “no” based on whether they were correct or incorrect according to scientifically accepted definitions (e.g., zoonoses were coded as correctly defined if described as “animal diseases that can infect humans”).

### 2.7. Ethical Consideration

Ethical clearance was obtained from the University of Johannesburg Research Ethics Committee (REC-1529-2022). Prior to data collection, formal permission to conduct the study was secured from the Director of Veterinary Services in the North West Province. Participation was voluntary, and written informed consent was obtained from all respondents. Confidentiality was maintained through anonymized data collection and secure storage of both physical and electronic records in password-protected devices and locked cabinets accessible only to the research team.

## 3. Results

### 3.1. Response Rate and Demographics

Out of 137 eligible veterinary fieldworkers, 105 completed the questionnaire, yielding a response rate of 76.6%. All general/farm aid workers declined to participate. Reasons for declining were not collected. As shown in Table 2, the majority of respondents were AHTs (76.2%, 80/105), with a near-equal distribution of males and females overall. The VPHOs were predominantly female (84.6%), while 75.0% of veterinarians were male, likely reflecting historical female-to-male dominance patterns in veterinary roles.

Most respondents (66.7%) were in the age category of 34–49 years and experienced, with over two-thirds (67.6%) having more than ten years of fieldwork, particularly among veterinarians (83.3%) and VPHOs (69.2%). This indicates a mature, experienced workforce across all occupational groups.

### 3.2. Occupational Health and Safety Knowledge by Job Category

As shown in Table 3, overall OHS training was low among participants. Most participants had not received departmental veterinary OHS training (79.0%), biosecurity and biosafety training (77.1%), or other OHS-related training (86.7%). However, veterinarians reported higher access to both departmental and external training compared to AHTs and VPHOs.

Despite limited formal training, participants demonstrated strong knowledge of zoonotic diseases, especially Rabies (88.6%) and *Brucella abortus* (81.0%). Interestingly, VPHOs showed better knowledge of *Listeria monocytogenes* in meat (92.3%) than the other groups. Veterinarians, surprisingly, had lower Rabies knowledge (88.6%) compared to AHTs (91.3%) and VPHOs (92.3%), suggesting potential training gaps even among more qualified staff.

### 3.3. Occupational Health and Safety Practices by Job Category

Table 4 summarizes PPE practices regarding its availability and usage. Overalls were more commonly available (90.5%) and the most consistently used (86.7%) item, with variation in usage among job categories. Safety shoes/boots were available to 83.8% but were only used by 76.2% of the respondents. The findings demonstrated a disconnect between the availability of PPE and its consistent use among the fieldworkers. For instance, while 91.7% of respondents reported that gloves were available, only 33.3% reported always using them. Similarly, although 66.7% had access to face masks, none indicated that they always used them.

Face shields were the least available and least used PPE.

Vaccination status was assessed through self-reported history of vaccinations received within the preceding five years. Only 38.1% of all participants were vaccinated against zoonotic diseases, with veterinarians having the highest vaccination rate (75%), followed by AHTs (38.8%). No VPHOs reported any vaccination, reflecting a critical gap in occupational health protection.

### 3.4. Zoonotic Exposure Risk Factors by Job Category

As shown in Table 5, most participants reported frequent exposure to Brucellosis-infected animals/materials (69.5%), other potentially infectious materials (82.9%), animal vaccines (80.0%), and sharps injuries (74.3%). These exposures were significantly more common among AHTs and veterinarians (*p* < 0.001), aligning with their hands-on roles in fieldwork. Exposure to Rabies (20.0%), Bovine Tuberculosis (12.4%), and Anthrax (16.2%) was less frequent, with no significant differences between occupational groups. The self-reported history of zoonotic infection was low (5.7%), with all cases occurring among AHTs.

### 3.5. Perceptions of OHS Improvements Among Participants

Table 6 outlines participant perceptions of OHS improvements to prevent zoonotic diseases. The majority of AHTs (67.5%) and VPHOs (53.8%) believed that OHS knowledge required improvement, compared to only 35.0% of veterinarians. A similar pattern was observed regarding the need for improved OHS/biosecurity training, with 75% of AHTs and 92.3% of VPHOs indicating this need, versus only 33.3% of veterinarians. Across all job categories, the highest-rated areas for improvement included knowledge of OHS policies (84.8%) and access to training (72.4%). PPE availability was perceived as inadequate by 68.8% of AHTs and 50.0% of veterinarians, although VPHOs were less concerned.

## 4. Discussion

The current study provides important insights into OHS knowledge, practices, and zoonotic risk exposures among veterinary fieldworkers in the North West Province, South Africa. The findings demonstrate significant disparities in OHS knowledge and training across three job categories (i.e., AHTs, Vets, and VPHOs), with implications for targeted interventions.

The distribution and dominance of mid-career professionals (aged 34–49) indicate the need for strategic succession planning to attract younger professionals into the field. Despite most participants being experienced, a concerning proportion reported not receiving departmental OHS training. Notably, AHTs and VPHOs, who play important roles in handling biological materials, were less likely to have received biosecurity and biosafety training than Vets. This gap is concerning, as inadequate training may result in unsafe practices that contribute to the spread of zoonotic diseases in this area of work. The necessity for biosecurity knowledge and practices is well-documented in the literature. Studies and systematic reviews on biosecurity in agriculture suggest that biosecurity knowledge and application of interventions protect animal health and directly reduce the risk of zoonotic pathogens transmission to humans [11,20,21].

Although formal training was lacking for many, VPHOs demonstrated better knowledge in specific zoonotic scenarios, particularly those related to OHS, possibly due to their academic curriculum. This observation echoes previous studies emphasizing the integration of occupational hazard content in veterinary education to enhance awareness and safety practices [22,23].

Regarding PPE, participants acknowledged its importance, yet usage varied by job category and PPE type. Veterinarians used overalls and gloves less often despite their availability. These findings are similar to those found in an Arizona study, which found that 37% of veterinarians used PPE while handling clinically ill animals and an even lower 17% while handling healthy animals [13]. This could be attributed to their lower risk perception, attributed to their higher level of vaccinations. While overalls and safety shoes were consistently used, gloves and face masks were less frequently worn, despite availability, pointing to behavioral and policy gaps. Face mask use showed the most variation, suggesting differences in perceived risk or compliance culture across the three roles. These discrepancies suggest that access alone is insufficient; PPE use must be aligned with risk-specific tasks and reinforced through regular training and policy enforcement [24,25,26,27]. Risk-based PPE deployment is critical, especially considering that practices differ between abattoirs and field settings. For instance, hand hygiene may be more appropriate than gloves in abattoir environments, as per local regulations [28]. The variation in variables (e.g., PPE use, vaccination status) suggests the need for targeted training and monitoring interventions by job category.

The self-reported zoonotic infection prevalence (5.7%) was lower than anticipated, considering that over 70% of participants reported regular exposure to risk factors. The discrepancy should be interpreted with caution, as several factors may explain this finding, including recall bias and under-recognition of mild or asymptomatic infections inherent in self-reported data, as well as the potential protective effects of vaccination, acquired immunity, or use of PPE that may reduce symptomatic disease despite exposure. Structural health system limitations, such as restricted diagnostic capacity and frequent misattribution of zoonotic diseases to other causes, may also contribute to underreporting. Furthermore, the cross-sectional design captured point prevalence rather than cumulative incidence over participants’ work histories, which may have likely led to underestimation of the true burden. These factors highlight both the limitations of self-reported measures and the need for future studies incorporating serological testing or clinical surveillance to better estimate the actual prevalence of zoonotic infections [5,7,21]. Given that veterinarians had the highest vaccination coverage and the lowest infection rate, expanding vaccination access to AHTs and VPHOs may be a key protective strategy. The poor vaccination coverage signals an urgent need for occupational health policy enforcement.

More than half the respondents highlighted a need for improvement in OHS training, policies, and PPE provision. AHTs and VPHOs were particularly vocal, suggesting potential implementation gaps in these groups. This aligns with global studies emphasizing the role of continuous professional development in reducing occupational infections [16,17].

### Study Limitation

The study limitations included participants consisting only of professional fieldworkers, who all had tertiary qualifications as general/farm aid workers, who declined to participate in the voluntary study. Due to limited resources, in-person meetings could not be conducted. Furthermore, participants could decline or withdraw from the study without providing any reason as per institutional ethics guidelines. Thus, the results should be interpreted with circumspection, as education level could not be assessed as contributing to OHS knowledge and practices. The study might have also been limited by recall or selective bias, particularly for the self-reported prevalence of zoonotic infection, where they might not have been able to recall zoonotic infections or be selective in their recall. Additionally, the relatively small sample sizes of the VPHOs and Vets compared to the AHTs limited the analyses. Thus, only descriptive results are presented. In addition, several key questions that may have affected the exposure risk were not included, such as the frequency, duration, and type of animal exposure. The study was only conducted in one province; therefore, the findings are not generalizable to other South African provinces, warranting the need for further research.

## 5. Conclusions

This study demonstrates the need to strengthen OHS systems within veterinary services in South Africa. Gaps in biosecurity and biosafety training, inadequate PPE usage, and variable awareness of zoonotic disease risks highlight areas requiring urgent attention. Tailored interventions, especially for AHTs and VPHOs, are essential to mitigate occupational exposure and protect both workers and the general public. Although the overall infection prevalence was low, this does not necessarily reflect low risk. Differences in vaccination uptake, underreporting, and inconsistent OHS practices necessitate a more integrated monitoring and training approach to build occupational resilience in veterinary fieldworkers.

## 6. Recommendations

1. Implement a risk-based OHS programme that identifies job-specific exposure and apply the hierarchy of controls to ensure proper use of PPE and vaccination against occupational zoonoses.
2. Provide continuous training in OHS, biosecurity, and biosafety tailored to each job category. This should include zoonotic disease recognition and human symptoms to facilitate early intervention and reporting.
3. Encourage reporting of exposures, accidents, and symptoms among fieldworkers. Establish a cross-sectoral One Health surveillance system to monitor priority zoonoses like Brucellosis, Rabies, and Avian Influenza.
4. Ensure equitable distribution of appropriate PPE and access to recommended vaccinations, especially for AHTs and VPHOs, guided by task-specific risk assessments.
5. Strengthen undergraduate and postgraduate training by embedding occupational health and zoonotic disease modules to build foundational awareness among future professionals.

## Figures and Tables

**Table 1:** Roles and responsibilities of veterinary fieldworkers.

Occupational Category	Responsibility
State Veterinarians	Are responsible for managing and controlling animal diseases, conducting ante- and post-mortem inspections, and implementing biosafety and biosecurity protocols.
Animal Health Technicians (AHTs)	Support veterinarians by collecting diagnostic specimens, administering vaccines, and participating in disease surveillance programs.
General/farm aid workers	Assist with animal and equipment handling and cleaning
Veterinary Public Health Officers (VPHOs)	Oversee food safety, animal welfare, and hygiene compliance in abattoirs, and are also involved in sample collection and inspection activities.

**Table 2:** Demographic characteristics of the participants by job category.

Variable	AHT n (%)	VPHO n (%)	Vet n (%)	Total N (%)
Sex				
Female	38 (47.5)	11 (84.6)	3 (25.0)	52 (49.5)
Male	42 (52.5)	2 (15.4)	9 (75.0)	53 (50.5)
Age				
18–33	13 (16.2)	2 (15.4)	1 (8.3)	16 (15.2)
34–49	52 (65.0)	9 (69.2)	9 (75.0)	70 (66.7)
50–65	15 (18.8)	2 (15.4)	2 (16.7)	19 (18.1)
Years of fieldwork experience				
0–5 years	19 (23.8)	0 (0.0)	1 (8.3)	20 (19.0)
6–10 years	9 (11.2)	4 (30.8)	1 (8.3)	14 (13.4)
>10 years	52 (65.0)	9 (69.2)	10 (83.4)	71 (67.6)

Key: AHT—animal health technician, VPHO—veterinary public health officer, Vet—veterinarian.

**Table 3:** Occupational health and safety knowledge by job category.

Variable	AHT n (%)	VPHO n (%)	Vet n (%)	Total N (%)
Departmental veterinary OHS training				
Yes	17 (21.2)	2 (15.4)	3 (25.0)	22 (21.0)
No	63 (78.8)	11 (84.6)	9 (75.0)	83 (79.0)
Departmental biosecurity & biosafety training				
Yes	15 (18.8)	2 (15.4)	7 (58.3)	24 (22.9)
No	65 (81.2)	11 (84.6)	5 (41.7)	81 (77.1)
Other OHS/biosecurity/biosafety training				
Yes	9 (11.2)	0 (0.0)	5 (41.7)	14 (13.3)
No	71 (88.8)	13 (100.0)	7 (58.3)	91 (86.7)
Accurate knowledge of what zoonosis is				
Yes	75 (93.8)	13 (100.0)	11 (91.7)	99 (94.3)
No	5 (6.2)	0 (0)	1 (8.3)	6 (5.7)
Accurate knowledge of *Brucella abortus*				
Yes	67 (83.8)	10 (76.9)	8 (66.7)	85 (81.0)
No	13 (16.3)	3 (23.1)	4 (33.3)	20 (19.0)
Accurate knowledge of rabies				
Yes	73 (91.2)	12 (92.3)	8 (66.7)	93 (88.6)
No	7 (8.8)	1 (7.7)	4 (33.3)	12 (11.4)
Accurate knowledge of the risk of a syringe with live Brucellosis vaccine				
Yes	51 (63.7)	9 (69.2)	11 (91.7)	71 (67.6)
No	29 (36.3)	4 (30.8)	1 (8.3)	34 (32.4)
Accurate knowledge of *Listeria Monocytogenes* in meat				
Yes	40 (50.0)	12 (92.3)	9 (75.0)	61 (58.1)
No	40 (50.0)	1 (7.7)	3 (25.0)	44 (41.9)
Knowledge of OHS/biosecurity/biosafety policies				
Yes	14 (17.5)	2 (15.4)	4 (33.3)	20 (19.0)
No	66 (82.5)	11 (84.6)	8 (66.7)	85 (81.0)
Knowledge of precautionary measures				
Yes	69 (86.2)	9 (69.2)	12 (100.0)	90 (85.7)
No	11 (13.8)	4 (30.8)	0 (0.0)	15 (14.3)
Knowledge of the importance of using PPE				
Agree	79 (98.8)	12 (92.3)	11 (91.7)	102 (91.1)
Disagree	1 (1.2)	1 (7.7)	1 (8.3)	3 (2.9)

Key: AHT—animal health technician, VPHO—veterinary public health officer, Vet—veterinarian.

**Table 4:** Occupational health and safety practices by job category.

Variable	AHT n (%)	VPHO n (%)	Vet n (%)	Total N (%)
Overalls available				
Yes	71 (88.8)	12 (92.3)	12 (100.0)	95 (90.5)
No	9 (11.2)	1 (7.7)	0 (0.0)	10 (9.5)
Overalls use				
Always	72 (90.0)	12 (92.3)	7 (58.3)	91 (86.7)
Sometimes	6 (7.5)	1 (7.7)	5 (41.7)	12 (11.3)
Never	2(2.5)	0 (0.0)	0 (0.0)	2 (2.0)
Safety shoes/boots available				
Yes	66 (82.5)	12 (92.3)	10 (83.3)	88 (83.8)
No	14 (17.5)	1 (7.7)	2 (16.7)	17 (16.2)
Safety shoes/boots use				
Always	59 (73.7)	11 (84.6)	10 (83.3)	80 (76.2)
Sometimes	13 (16.3)	1 (7.7)	2 (16.7)	16 (15.2)
Never	8 (10.0)	1 (7.7)	0 (0.0)	9 (8.6)
Gloves available				
Yes	61 (76.3)	4 (30.8)	11 (91.7)	76 (72.4)
No	19 (23.7)	9 (69.2)	1 (8.3)	29 (27.6)
Gloves use				
Always	30 (37.5)	2 (15.4)	4 (33.3)	36 (34.3)
Sometimes	44 (55.0)	8 (61.5)	8 (66.7)	60 (57.1)
Never	6 (7.5)	3 (23.1)	0 (0.0)	9 (8.6)
Face mask available				
Yes	44 (55.0)	11 (84.6)	8 (66.7)	63 (60.0)
No	36 (45.0)	2 (15.4)	4 (33.3)	42 (40.0)
Face mask use				
Always	26 (32.4)	11 (84.6)	0 (0.0)	37 (35.2)
Sometimes	27 (33.8)	1 (7.7)	9 (75.0)	37 (35.2)
Never	27 (33.8)	1 (7.7)	3 (25.0)	31 (29.6)
Face shield available				
Yes	22 (27.5)	3 (23.1)	3 (25.0)	28 (26.7)
No	58 (72.5)	10 (76.9)	9 (75.0)	77 (73.3)
Face shield use				
Always	4 (5.0)	0 (0.0)	0 (0.0)	4 (3.8)
Sometimes	26 (32.5)	1 (7.7)	4 (33.3)	31 (29.5)
Never	50 (62.5)	12 (92.3)	8 (66.7)	70 (66.7)
Zoonotic diseases vaccination status				
Vaccinated	31 (38.8)	0 (0.0)	9 (75.0)	40 (38.1)
Not vaccinated	49 (61.2)	13 (100.0)	3 (25.0)	65 (61.9)

Key: AHT—animal health technician, VPHO—veterinary public health officer, Vet—veterinarian.

**Table 5:** Analysis of zoonotic exposure risk factors among study participants by job category.

Variable	AHT n (%)	VPHO n (%)	Vet n (%)	Total n (%)
Exposure to animals/material with Brucellosis				
Often	63 (78.8)	2 (15.4)	8 (66.7)	73 (69.5)
Not often	17 (21.2)	11 (84.6)	4 (33.3)	32 (30.5)
Exposure to animals/materials with Rabies				
Often	17 (21.2)	1 (7.7)	3 (25.0)	21 (20.0)
Not often	63 (78.8)	12 (92.3)	9 (75.0)	84 (80.0)
Exposure to animals/material with Bovine Tuberculosis				
Often	10 (12.5)	2 (15.4)	1 (8.3)	13 (12.4)
Not often	70 (87.5)	11 (84.6)	11 (91.7)	92 (87.6)
Exposure to animals/material with Anthrax				
Often	14 (17.5)	2 (15.4)	1 (8.2)	17 (16.2)
Not often	66 (82.5)	11 (84.6)	11 (91.7)	88 (83.8)
Exposure to animal vaccines				
Often	74 (92.5)	2 (15.4)	8 (66.7)	84 (80.0)
Not often	6 (7.5)	11 (84.6)	4 (33.3)	21 (20.0)
Exposure to other potentially infectious materials/animals				
Often	72 (90.0)	6 (46.2)	9 (75.0)	87 (82.9)
Not often	8 (10.0)	7 (53.8)	3 (25)	18 (17.1)
Exposure to sharps injuries (needle pricks, blade cuts, etc.)				
Often	65 (81.2)	4 (30.8)	9 (75.0)	78 (74.3)
Not often	15 (18.8)	9 (69.2)	3 (25.0)	27 (25.7)
Zoonotic disease infection				
Yes	6(7.5)	0 (0.0)	0 (0.0)	6 (5.7)
No	74 (92.5)	13 (100)	12 (100)	99 (94.3)

Key: AHT—animal health technician, VPHO—veterinary public health officer, Vet—veterinarian.

**Table 6:** Comparison of participant perceptions of their need for OHS improvements by job category.

Variable	AHT (n = 80) n (%)	VPHO (n = 13) n (%)	Vet (n = 12) n (%)	Total (n = 105) N (%)
Need improvement in OHS knowledge				
Yes	54 (67.5)	7 (53.8)	3 (35.0)	64 (61.0)
No	26 (32.5)	6 (46.2)	9 (65.0)	41 (39.0)
Need improvement in biosecurity/biosafety knowledge				
Yes	48 (60.0)	8 (61.5)	3 (25.0)	59 (56.2)
No	32 (40.0)	5 (38.5)	9 (75.0)	46 (43.8)
Need improvement in OHS practices to prevent zoonotic disease infections				
Yes	43 (53.8)	8 (61.5)	3 (25.0)	54 (51.4)
No	37 (46.2)	5 (38.5)	9 (75.0)	51 (48.6)
Need improvement in OHS, biosecurity/biosafety training				
Yes	60 (75.0)	12 (92.3)	4 (33.3)	76 (72.4)
No	20 (25.0)	1 (7.7)	8 (66.7)	29 (27.6)
Need improvement in OHS, biosecurity/biosafety policies and procedures				
Yes	69 (86.2)	12 (92.3)	8 (66.7)	89 (84.8)
No	11 (13.8)	1 (7.7)	4 (33.3)	16 (15.2)
Need improvement in PPE availability				
Yes	55 (68.8)	5 (38.5)	6 (50.0)	66 (62.9)
No	25 (31.2)	8 (61.5)	6 (50.0)	39 (37.1)

Key: AHT—animal health technician, VPHO—veterinary public health officer, Vet—veterinarian.

## Data Availability

Data are available upon request and within the prescripts of the Protection of Personal Information Act (POPIAct).

## Abbreviations

The following abbreviations are used in this manuscript:

AHT	Animal health technicians
OHS	Occupational health and safety
PPE	Personal protective equipment
UK	United Kingdom
USA	United States of America
Vets	Veterinarians
VPHOs	Veterinary public health officers

## References

[1] World Health Organization (WHO) (2020). Zoonoses. https://www.who.int/news-room/fact-sheets/detail/zoonoses.

[2] Magwedere K., Hemberger M.Y., Hoffman L.C., Dziva F. (2012). Zoonoses: A potential obstacle to the growing wildlife industry of Namibia. Infect. Ecol. Epidemiol..

[3] Nguyen T.T., Mai T.N., Dang-Xuan S., Nguyen-Viet H., Unger F., Lee H.S. (2024). Emerging zoonotic diseases in Southeast Asia in the period 2011–2022: A systematic literature review. Vet. Q..

[4] Centre for Disease Control and Prevention (CDC) (2019). Zoonotic Diseases. https://www.cdc.gov/onehealth/basics/zoonotic-diseases.html.

[5] Newmana H., Shareef A. (2018). Zoonotic viral infections in South Africa: An overview. Res. Rev. Insights.

[6] Mobo B.H.P., Rabinowitz P.M., Conti L.A., Taiwo O.A. (2010). Occupational Health of Animal Workers. Hum. Anim. Med..

[7] World Health Organization (WHO) (2024). Occupational Health. https://www.who.int/health-topics/occupational-health.

[8] Gummow B.A. (2003). Survey of zoonotic diseases contracted by South African veterinarians. J. S. Afr. Vet. Assoc..

[9] World Organization for Animal Health (WOAH) (2018). Terrestrial Code Online Access. https://www.woah.org/fileadmin/Home/fr/Health_standards/tahm/1.01.04_BIOSAFETY_BIOSECURITY.pdf.

[10] De Cooman C. (2022). Safe Handling and Biosecurity Measures. https://www.agproud.com/articles/56210-safe-animal-handling-and-biosecurity-measures.

[11] Msimang V., Rostal M.K., Cordel C., Machalaba C., Tempia S., Bagge W., Burt F.J., Karesh W.B., Paweska J.T., Thompson P.N. (2022). Factors affecting the use of biosecurity measures for the protection of ruminant livestock and farm workers against infectious diseases in central South Africa. Transbound. Emerg. Dis..

[12] Simpson G., Quesada F., Chatterjee P., Kakkar M., Chersich M.F., Thys S. (2021). Research priorities for control of zoonoses in South Africa. Trans. R. Soc. Trop. Med. Hyg..

[13] Venkat H., Yaglom H.D., Adams L. (2019). Knowledge, attitudes, and practices relevant to zoonotic disease reporting and infection prevention practices among veterinarians—Arizona, 2015. Prev. Vet. Med..

[14] D’Souza E., Barraclough R., Fishwick D., Curran A. (2009). Management of occupational health risks in small-animal veterinary practices. Occup. Med..

[15] McLean M., Watson H., Muswema A. (2007). Veterinary waste disposal: Practice and policy in Durban, South Africa (2001–2003). J. Waste Manag..

[16] World Health Organization (WHO) (2020). Control of Neglected Tropical Diseases. https://www.who.int/teams/control-of-neglected-tropical-diseases/rabies/.

[17] National Department of Health, National Institute for Communicable Diseases (2021). National Guidelines for the Prevention of Rabies in Humans, South Africa. https://www.nicd.ac.za/wp-content/uploads/2021/08/Human-Rabies-Prophylaxis-Guideline-For-South-Africa_27-August-2021.pdf.

[18] Department of Agriculture Land Reform and Rural Development (2021). Animal Disease Reporting January–December 2021. https://www.nda.gov.za/index.php/publication/430-disease-reporting.

[19] Guerin R.J., Sleet D.A. (2020). Using Behavioral Theory to Enhance Occupational Safety and Health: Applications to Health Care Workers. Am. J. Lifestyle Med..

[20] Kimman T., Hoek M., de Jong M.C. (2013). Assessing and controllinghealth risks from animal husbandry. NJAS Wagening. J. Life Sci..

[21] Layton D.S., Choudhary A., Bean A.G. (2017). Breaking the chain of zoonoses through biosecurity in livestock. Vaccine.

[22] Landge S., Tripathi H., Agarwal R.K., Banthiya V. (2022). Knowledge level of veterinarians about occupational health hazards and the constraints felt by them for safe and hazard free working conditions. J. Vet. Public Health.

[23] Jayanthi R., Boopathy R.M. (2021). Occupational Health Hazards in Veterinarians—A Review. Pharma Innov. J..

[24] Centre for Disease Control and Prevention (CDC) (2018). Veterinary Safety & Health: Hazard Prevention and Infection Control. https://www.cdc.gov/niosh/topics/veterinary/hazard.html.

[25] Wright J.G., Jung S., Holman R.C., Marano N.N., McQuiston J.H. (2008). Infection Control Practices and Zoonotic Disease Risks Among Veterinarians in the United States. J. Am. Vet. Med. Assoc..

[26] Business Queensland (2018). Veterinary Use of Personal Protective Equipment and Disinfectants. https://www.business.qld.gov.au/industries/service-industries-professionals/service-industries/veterinary-surgeons/guidelines-hendra/ppe-disinfectants.

[27] Robin C., Bettridge J., McMaster F. (2017). Zoonotic disease risk perceptions in the British veterinary profession. Prev. Vet. Med..

[28] Christmann U. (2020). Best practices in veterinary personal protective equipment. Rev. Sci. Tech..

